# Decoding Selective Attention in Normal Hearing Listeners and Bilateral Cochlear Implant Users With Concealed Ear EEG

**DOI:** 10.3389/fnins.2019.00720

**Published:** 2019-07-18

**Authors:** Waldo Nogueira, Hanna Dolhopiatenko, Irina Schierholz, Andreas Büchner, Bojana Mirkovic, Martin G. Bleichner, Stefan Debener

**Affiliations:** ^1^Department of Otolaryngology, Hearing4all, Hannover Medical School, Hanover, Germany; ^2^Neuropsychology Lab, Department of Psychology, Hearing4all, Carl von Ossietzky University of Oldenburg, Oldenburg, Germany

**Keywords:** cochlear implant, EEG, cEEGrid, selective attention, speech decoding

## Abstract

Electroencephalography (EEG) data can be used to decode an attended speech source in normal-hearing (NH) listeners using high-density EEG caps, as well as around-the-ear EEG devices. The technology may find application in identifying the target speaker in a cocktail party like scenario and steer speech enhancement algorithms in cochlear implants (CIs). However, the worse spectral resolution and the electrical artifacts introduced by a CI may limit the applicability of this approach to CI users. The goal of this study was to investigate whether selective attention can be decoded in CI users using an around-the-ear EEG system (cEEGrid). The performances of high-density cap EEG recordings and cEEGrid EEG recordings were compared in a selective attention paradigm using an envelope tracking algorithm. Speech from two audio books was presented through insert earphones to NH listeners and via direct audio cable to the CI users. 10 NH listeners and 10 bilateral CI users participated in the study. Participants were instructed to attend to one out of the two concurrent speech streams while data were recorded by a 96-channel scalp EEG and an 18-channel cEEGrid setup simultaneously. Reconstruction performance was evaluated by means of parametric correlations between the reconstructed speech and both, the envelope of the attended and the unattended speech stream. Results confirm the feasibility to decode selective attention by means of single-trial EEG data in NH and CI users using a high-density EEG. All NH listeners and 9 out of 10 CI achieved high decoding accuracies. The cEEGrid was successful in decoding selective attention in 5 out of 10 NH listeners. The same result was obtained for CI users.

## Introduction

Cochlear implants (CIs) are medical devices that partly replace the function of a damaged inner ear. It roughly consists of a behind-the-ear sound processor and a set of electrode contacts located inside the cochlea. The CI acts as a kind of artificial cochlea, transforming the acoustic signals, captured by a microphone into electric pulses, thereby bypassing the damaged structures of the ear and directly stimulating the auditory nerve (e.g., Wilson and Dorman, 2008). Most CI users obtain good speech understanding in the absence of background noise (Krueger et al., 2008; Zeng et al., 2008). However, CI users still face difficulties in understanding speech in more challenging listening environments with multiple speakers, background noise and reverberation (i.e., the cocktail party problem; Cherry, 1953). In such situations, normal-hearing (NH) listeners can focus on one target speaker and effectively suppress other present speakers (Mesgarani and Chang, 2012). This ability is probably impaired in CI users and may be one of the reasons for the limitations in speech understanding in challenging listening environments. This work investigates the possibilities of decoding selective attention in CI users.

Auditory selective attention seems to operate via facilitation and inhibition mechanisms (e.g., Michie et al., 1993; Bidet-Caulet et al., 2007). It modulates low-frequency oscillations of cortical responses to speech stimuli, exhibiting both enhanced tracking of target-speech signals and enhanced suppression of masker-speech signals (e.g., Mesgarani and Chang, 2012; O’Sullivan et al., 2015). Neural activity in the cerebral cortex, especially in the delta (1–4 Hz) and theta (4–8 Hz) frequency bands, tracks the amplitude envelope of a complex auditory stimulus such as speech (Ding and Simon, 2012; Giraud and Poeppel, 2012; Power et al., 2012). It is now well accepted that auditory selective attention can modulate the sensory analysis of relevant and irrelevant stimuli not only in the auditory cortex (Coch et al., 2005; Bidet-Caulet et al., 2007), but also at the level of the brainstem (e.g., Forte et al., 2017) and even peripherally at the level of the cochlea as demonstrated by changes in otoacoustic emissions (e.g., Walsh et al., 2015).

Previous studies have shown that an attended target sound source can be identified from cortical recordings in demanding auditory scenarios. This has been proven using several recording technologies, such as electrocorticography (Nourski et al., 2009; Zion Golumbic et al., 2013), magnetoencephalography (Ding and Simon, 2012; Akram et al., 2016), and electroencephalography (EEG) (Mirkovic et al., 2015; O’Sullivan et al., 2015). O’Sullivan et al. (2015) showed that it is possible to detect an attended speech source from cortical activity in a simplistic cocktail party scenario. The procedure for detecting the target speaker was based on an envelope tracking approach, which correlates the speech envelope with the recorded cortical activity using a linear model. Previous studies, using a two-speaker scenario, reached a decoding accuracy of up to 88% based on single-trial EEG recordings in NH subjects (e.g., Mirkovic et al., 2015). Moreover, Wöstmann et al. (2016) showed that the power of alpha rhythm in subjects with hearing problems is higher than in people without any problems. Finally, a recent study by Nogueira et al. (2019) demonstrated that in principle it is possible to decode selective attention in CI users with an accuracy of up to 70% using high-density EEG recordings. The lower decoding accuracy compared to the results in NH listeners may be explained by the smeared representation of the sound and by the electrical artifacts of the CI introduced into the EEG recordings.

In general, best decoding results can be obtained using a high-quality and high-density EEG system. However, the idea to use real-time EEG recordings to report the current focus of attention requires the establishment of a miniaturized EEG registration system that is portable, small in size, comfortable in use and which receives EEG signals with high accuracy (Bleichner and Debener, 2017). With this purpose, the C-shaped cEEGrid sensor array, comprising 10 flex-printed miniaturized EEG sensors that can be placed around the ear was developed (Debener et al., 2015). The cEEGrid sensors are light and comfortable to wear, providing the possibility for long-term EEG recordings without discomfort for the user. The cEEGrid system requires the use of a small amount of contact gel, thus combining the advantages of the wet EEG sensor technology with the advantages of the dry sensor technology, avoiding disadvantages of both systems. Previous work already applied the cEEGrid in a selective attention paradigm in NH listeners and reached a decoding accuracy of up to 70% (Mirkovic et al., 2016).

CIs have been designed not only to stimulate the auditory nerve but also to record neural activity from the intracochlear electrodes. Accordingly, the CI offers implanted stimulators and a receiver that can be used to record diverse auditory responses (see McLaughlin et al., 2012), as short latency compound action potentials, auditory brainstem responses (1–10 ms) and mid-to-late cortical potentials (20–300 ms). However, current commercial CIs only offer short recording time buffers and therefore these recordings have been only conducted in laboratory conditions. Though, in the near future it is expected that CIs will be optimized to record neural activity from the intracochlear electrodes. To this end it is essential to assess the limitations in recording neural activity in CI users from electrodes located close to the ear. As the electrodes are located close to the transmitter coil as well as the CI electrodes in the cochlea, the electrical artifact may be enhanced, which, combined with the smeared neural representations of sounds received by CI users, might limit the possibility to decode selective attention in CI users.

In our previous work we showed that it is possible to decode selective attention in CI users using a high-density EEG system (Nogueira et al., 2019). In the present study our goal was to examine the practicability of an around-the-ear EEG to decode selective attention in CI users. The data revealed that, on single-subject level, it is possible to infer the listener’s focus of attention from single-trial EEG using scalp electrodes in both, NH and CI users. However, accuracy drops when only using around-the-ear EEG electrodes.

## Materials and Methods

### Participants

10 NH listeners (8 male; mean age: 42.8, range: 24–77, SD: 20.2 years) and 10 bilateral CI users (5 male; mean age: 67.5, range: 59–80, SD: 6.13 years) participated in the study. Speech recognition scores for both CI users and NH listeners were assessed using the Göttingen sentence test in adaptive background noise (Kollmeier and Wesselkamp, 1997). CI users furthermore performed the Hochmair-Schulz-Moser sentence test in noise (+10 dB SNR; Hochmair-Desoyer et al., 1997). All subjects were native German speakers. CI users had more than 6 months experience with their implant. The demographics for NH listeners and CI users are provided in Tables 1, 2, respectively. Prior to the experiment all participants provided written informed consent and the study was carried out in accordance with the Declaration of Helsinki principles, approved by the Ethics Committee of the Hannover Medical School.

**TABLE 1:** Subject demographics NH listeners.

***#***	**Göttingen sentence test (dB SNR for SRT50%)**	**Attended story**
NH1	−4.8	Jules Verne
NH2	−6.6	Brother Grimm
NH3	−6.0	Brother Grimm
NH4	−5.6	Jules Verne
NH5	−5.4	Jules Verne
NH6	−5.6	Brother Grimm
NH7	−4.3	Jules Verne
NH8	−5.0	Brother Grimm
NH9	−3.8	Jules Verne
NH10	−3.2	Jules Verne

*F, female; M, male; GOESA, Göttingen sentence test in adaptive noise. Speech recognition.*

**TABLE 2:** Subject demographics CI users.

**#**	**Etiology**	**Age at** **onset of** **profound** **deafness** **(years;** L/R^*^)	Duration of deafness (months; L/R^*^)	Implant use (months; L/R^*^)	**HSM** **sentence test in noise (+10 dB SNR) (%)**	**Göttingen sentence test (dB SNR for SRT50%)**	**Attended story**
CI1	Genetic	59/25	1/265	109/243	73.58	n.a.	Brother Grimm
CI2	Unknown	50/50	38/92	102/49	95.28	0	Jules Verne
CI3	Genetic	54/49	1/49	115/128	66.04	3.2	Brother Grimm
CI4	Unknown	57/59	1/1	58/34	80.19	1.4	Jules Verne
CI5	Unknown	71/67	1/25	97/123	80.00	10.8	Jules Verne
CI6	Unknown	60/60	21/4	114/132	85.85	8.6	Brother Grimm
CI7	Genetic	69/71	1/1	33/12	50.00	7.4	Jules Verne
CI8	Unknown	56/56	60/47	38/71	78.30	2.6	Brother Grimm
CI9	Acute hearing loss	57/41	1/182	42186	83.96	11.9	Brother Grimm
CI10	Otosclerosis cochleae	33/50	289/20	112/178	89.62	1.6	Jules Verne

F, female; M, male. Age at onset of profound deafness refers to the age at which the amount of hearing loss was too severe to be sucessfully treated by a conventional hearing aid. Duration of deafness is defined as the time between the age at onset of profound deafness and the CI implantation.^*^Values are provided for the left and the right CI, respectively. HSM, Hochmair-Schulz-Moser sentence test (+10 dB SNR). GOESA, Göttingen sentence test in adaptive noise. Speech recognition scores are obtained in a bilateral listening condition.

### Stimuli

Similar to previous studies (Mirkovic et al., 2015; Nogueira et al., 2019) two German narrations (‘A drama in the air’ by Jules Verne narrated by a male speaker and ‘Two brothers’ by the Grimm brothers, narrated by a female speaker) were presented to the study participants. To maintain attention to the correct story and to avoid capture of attention by the other story, silent periods were limited to 0.5 s. The audio signal for NH was provided through inserted earphones (3M E-A-RTONE 3A, 50 ohm). CI users received the audio signal via audio cables directly connecting the sound system and the CI sound processor. Stimulus presentation was controlled by the Presentation Software (Neurobehavioral Systems, Inc., Berkeley, CA, United States; version 16.5) as in our previous work (Nogueira et al., 2019). Every participant adjusted the loudness to an individual moderate level (60–70 dB(A); Allen et al., 1990; Zeng, 1994) by means of a seven-point loudness-rating scale (where 1 is “very soft” and 7 is “extremely loud”).

### Procedure

Each participant was instructed to sit relaxed and calm, keep eyes open, and maintain fixation to a fixation point on a screen in the front. The audio stories were segmented into 24 parts of 2 min each, resulting in a total stimulus presentation time of 48 min. The whole paradigm was subdivided into 6 sections with 4 segments each. After each section participants had a break. Stories were presented simultaneously, with one story being presented at the one and one story being presented at the other ear. Subjects were instructed to focus their attention to one of the two concurrent stories, while ignoring the other one. The to-be-attended story was randomized between participants. Each participant, respectively, attended to the same story throughout the whole session, but the side on which the attended speech stream was presented changed after each 2 min segment to exclude effects of side of presentation. Before the start of each segment, participants were instructed which side to attend. The starting side of the attended speech stream was randomized between participants. After each 2 min segment, participants had to answer eight multiple-choice questions, four related to the attended and four to the unattended story, with four answer possibilities each, to ensure attention to the correct story.

### EEG Recordings

The recording of the EEG data was performed in an electromagnetically and acoustically shielded booth. High-density continuous EEG data were recorded using a BrainAmp System (Brain Products GmbH, Gilching, Germany) with 96 Ag/AgCl electrodes mounted in a customized, infracerebral electrode cap with an equidistant electrode layout (Easycap GmbH, Herrsching, Germany). The ground electrode was placed on a central frontopolar site, and the nose tip served as reference. Data were recorded with a sampling rate of 1000 Hz and an online filter from 0.2 to 250 Hz. Impedances were controlled and maintained below 10 kΩ. Some electrodes around the ears and in the proximity of the transmitter coil were excluded as a consequence of using the cEEGrid system or the placement of the CI. On average, across subjects, 8 electrodes were excluded.

**FIGURE 1:**
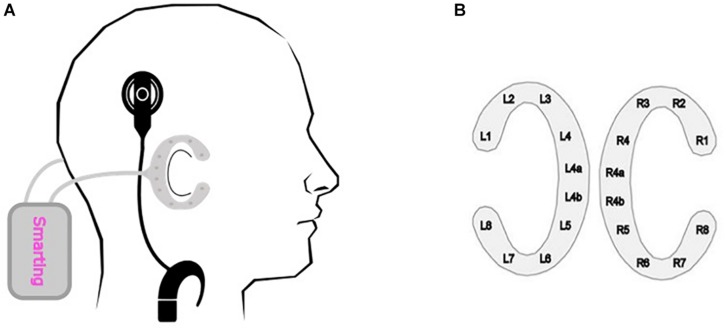
cEEGrid recording. **(A)** Schematical placement of the cEEGrid system. The C-shaped cEEGrid arrays are fixated around the ear with a double-sided adhesive. Both cEEGrid arrays are connected to a wireless mobile 24-channel EEG amplifier (SMARTING, mBrainTrain, Belgrade, Serbia). The CI sound processors were moved from their usual position (behind the ear) to the participant’s collar, as otherwise the CI processors would have occupied the same position as the cEEGrid. **(B)** cEEGrid electrode layout. Each cEEGrid array consists of 10 single electrodes. Electrodes L1–L4 and L5–L8 around the left ear and electrodes R1–R4 and R5–R8 around the right ear measure the voltage between the respective electrode and the reference electrode R4b. Electrode R4a is the ground electrode. The electrode L4b was used in the offline analysis for re-referencing the data. In order to keep the layout symmetric, electrode L4a was excluded from the data analysis. Accordingly, 16 channels, 8 around each ear, were used for the analysis.

The cEEGrid array contains 10 electrodes printed with silver ink on a flexible material that is placed around the ear (Debener et al., 2015). Before applying the cEEGrid system - one array to each side of the head – the skin behind the ear is prepared with an abrasive gel and alcohol. Subsequently, the cEEGrid arrays are placed around the ear with a double-sided adhesive, whereby the electrodes are covered with a small amount of electrolyte gel. The two cEEGrid arrays were connected to a wireless mobile 24-channel EEG amplifier (SMARTING, mBrainTrain, Belgrade, Serbia). Positions R4a and R4b were used as ground and reference, respectively (Figure 1). In the end, data from 18 electrodes were recorded with a 24 bit resolution and a 500 Hz sampling rate.

The CI sound processors were moved from their usual position (behind the ear) to the participant’s collar, as otherwise the CI processors would have occupied the same position as the cEEGrid. The change in placement did not affect the participant’s sound perception with the CI, as the sound was delivered via a direct audio cable and not received via the microphones.

**FIGURE 2:**
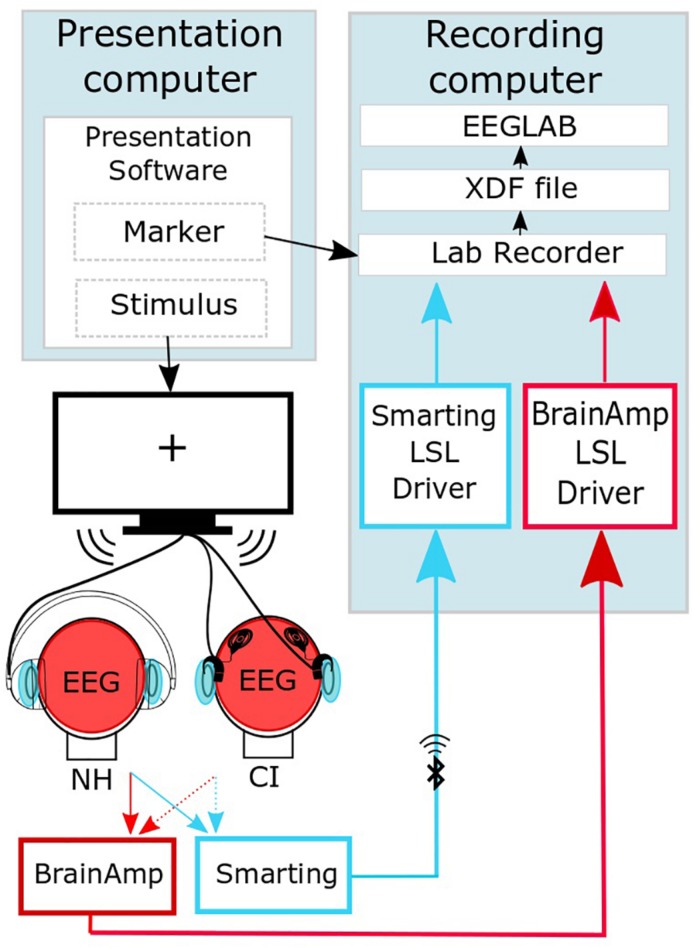
Synchronized streaming of the two separate EEG systems (adapted from Mirkovic et al., 2016). The software Presentation (Neurobehavioral Systems, Inc., Berkeley, CA, United States) was used to present the sound to the participants and to send event markers to the Lab recorder software. EEG was recorded with one cEEGrid located around each ear (head, light blue) and a high-density cap (head, red). By means of the SMARTING mobile amplifier (mBrainTrain, Belgrade, Serbia), the cEEGrid signal was wirelessly sent to the recording computer, while the cap-EEG signals were recorded using the Brain Amp amplifiers (BrainProducts GmbH, Gilching, Germany) with a physical connection to the recording computer. EEG streams were joined into one output file in an extensible data format (.xdf). Further preprocessing of the EEG data was performed using the EEGLAB Toolbox (Delorme and Makeig, 2004).

The data recorded with the high-density EEG cap were transmitted to a personal computer though a physical connection, whereas the data recorded with the cEEGrid arrays were transferred via Bluetooth. EEG streams were joined into one output file in an extensible data format (.xdf) using the LabStreamingLayer software^1^. The data were preprocessed offline using EEGLAB (Figure 2; Delorme and Makeig, 2004).

A timing test was performed to test for a delay between sound presentation and EEG recordings based on the procedure of Mirkovic et al. (2016). A time delay of 77 ms and 5 ms was observed for the BrainAmp and the SMARTING system, respectively. These time delays are corrected for the analysis in the results section.

### EEG and Speech Envelope Preprocessing

EEG data were analyzed offline using custom scripts in MATLAB 8.1.0.604 (R2013a; Mathworks, Natick, MA, United States) and EEGLAB version 14.0.0b (Delorme and Makeig, 2004). The obtained EEG data were preprocessed according to the procedure of similar previous studies (Mirkovic et al., 2015; O’Sullivan et al., 2015). The EEG data were filtered using a bandpass filter from 2 to 8 Hz and down sampled to 64 Hz. Afterward, the data were epoched in consecutive 60 s intervals, resulting in 48 trials for each subject. The envelopes of both audio stories were obtained using the Hilbert transform of the original audio signal. The audio signal likewise was low-pass filtered (8 Hz) and down sampled to 64 Hz.

### Speech Reconstruction

Speech reconstruction from the EEG data was accomplished through the “backward” modeling of neural responses (mTRF; Crosse et al., 2016). The toolbox developed by Crosse et al. (2016) can, in general, use “backward“ and “forward“ linear modeling, based on a least square estimation between neural data and the stimulus. The “forward” model can be used to predict the neuronal impulse response to a continuous stimulus, thereby describing how the system generates or encodes information (Haufe et al., 2014). The analysis of such impulse response can then be likened to traditional analysis of event-related potentials (Lalor et al., 2009; Petersen et al., 2016; Di Liberto et al., 2018). “Backward” model however, is used to reconstruct the attended stimulus from neural data and by comparing this reconstruction to all available stimuli, it is possible to decode the locus of attention, which is exactly what this work focuses on.

The spatio-temporal filter, also termed “decoder,” at specific time-lags *l* = 0…*L*−1 and electrodes n is denoted by w_n,l_, and performs the linear mapping from the neural response back to the speech envelope. *L* refers to the index corresponding with the upper boundary of time lags. The maximum time lag was set to 510 ms and a decoder was created every 15 ms resulting in *L* = 35decoders. The neural response at time sample *k* = 0…*K*−1 of the electrode *n* = 0…*N*−1, is denoted as _*yn*_ [*k*]. The reconstructed attended signal is estimated as follows:

(1) $${\hat{x}}_{a,u}\,\left[k\right]=\sum_{n=0}^{N-1}\sum_{l=0}^{L-1}w_{n,l}\cdot y_{n}\,\left[k+\Delta +l\right],$$

where ${\hat{x}}_{a,u}\,\left[k\right]$ denotes the reconstructed attended or unattended signal at time sample *k* = 0…*K*−1, and Δ models the latency or lag.

During training, the decoder *W*_*a*_ is estimated using least squares error minimization between the Hilbert envelope extracted from the attended audio signal *x*_*a*_[*k*] and the reconstructed envelope ${\hat{x}}_{a}\,\left[k\right]$. To avoid overfitting, regularization is applied, using the norm of the coefficients:

(2) $$J_{R\,L\,S}\,\left(W_{a}\right)=E\,\left\{{\left|x_{a}\,\left[k\right]-W_{a}^{T}\,Y\,\left[k\right]\right|}^{2}\right\}+\lambda \,W_{a}^{T}\,W_{a},$$

with λ being the regularization parameter.

Minimizing *J*_*RLS*_(*W*_*a*_), with respect to the decoder coefficients leads to the following solution:

(3) $$W_{a}^{T}={\left(R_{x_{a}\,Y}+\lambda \,I\right)}^{-1}\cdot R_{Y\,Y},$$

where R_x_a_Y_ and R_*YY*_ are defined as follows

(4) $$R_{x_{a}\,Y}=\sum_{m=0}^{K}x_{a}\,\left[k\right]\,y\,\left[k+m\right],$$

(5) $$R_{Y\,Y}=\sum_{m=0}^{K}y\,\left[k\right]\,y\,\left[k+m\right].$$

Empirically, it was found that a regularization parameter of λ =  0.001 led to the highest accuracy for the NH group, for both, the high-density scalp data and the cEEGrid data. As CI users had different CIs with different stimulation settings, the regularization parameter was fitted individually for each subject to maximize the selective attention accuracy.

Selective attention was decoded comparing the correlation coefficient between the reconstructed attended and the original attended signal ($R_{x_{a}\,{\hat{x}}_{a}})$ with the correlation coefficient between the reconstructed attended and the original unattended signal ($R_{x_{u}\,{\hat{x}}_{a}})$. If ($R_{x_{a}\,{\hat{x}}_{a}})$ was larger than $R_{x_{u}\,{\hat{x}}_{a}})$, the attended speech stream was decoded correctly. This procedure was repeated for all 48 trials and the accuracy of the decoder was calculated as the number of times the signal was correctly decoded, divided by total number of trials. To improve the procedure, “leave-one-out” cross-validation was used. Each test-trial was evaluated using the (averaged) decoder obtained from the average of the decoders trained on every other trial.

## Results

With the goal of assessing the feasibility to decode selective attention in CI users using the cEEGrid, we first analyzed the effect of reducing the number of EEG scalp electrodes to approximate the cEEGrid layout. Next, we evaluated the influence of the electrical CI artifact on the decoding accuracy using a simple experimental model. Furthermore, the effect of the regularization parameter on the decoding accuracy was examined for both, NH listeners and CI users. The correlation coefficients between the original envelope of the audio signal across lags and the reconstructed signal from both, the scalp EEG and the cEEGrid data were analyzed in detail. Finally, the overall decoding accuracy using a generic regularization parameter and the optimized decoding accuracy using an individual regularization parameter for each subject was reviewed.

### Effect of Reducing the Number of Electrodes

As the cEEGrid system has only a small number of electrodes compared to the high-density EEG cap, we evaluated the effect of decreasing the number of electrodes and the influence of electrode placement. We therefore calculated the decoding accuracy for different combinations of scalp electrodes, also trying to simulate the placement of the cEEGrid electrode arrays. The decoding accuracy was compared using all scalp electrodes, using 19 electrodes on the top and 14 electrodes from the area behind the ear. It should be mentioned that we did not interpolate data, but simply chose electrodes in the area of interest.

**FIGURE 3:**
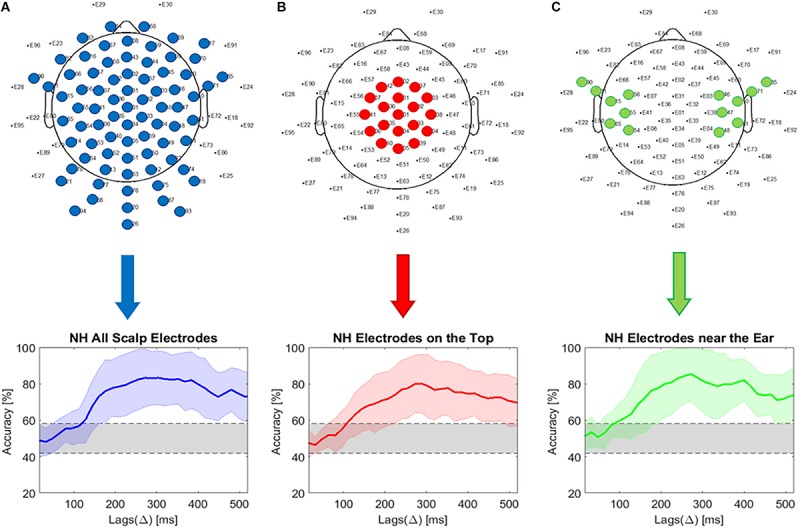
Comparison of electrode locations. **(A)** Selective attention decoding accuracy using all electrodes (eye electrodes 29 and 30 and scalp EEG electrodes spared at the position of the cEEGrid are excluded from the analysis); **(B)** Selective attention decoding accuracy using 19 electrodes on the top of scalp; **(C)** Decoding accuracy using 14 electrodes in the area next to the ear.

Figure 3A presents the decoding accuracy using all scalp EEG electrodes. It can be seen that the maximum accuracy of around 80% is obtained at a lag of around 320 ms. The chance level was estimated as 0.95 of the confidence interval of a binomial distribution (Combrisson and Jerbi, 2015) and resulted in a range between 41.8 and 58.2% and is presented as the shaded areas in Figure 3.

From the results presented in Figure 3B, it seems that as long as the number of electrodes is not reduced below 19 (for electrodes located on the top of the head) or 14 (for electrodes located close to the ears), the decoding accuracy remains similar. However, the chosen scalp electrodes are positioned higher than the cEEGrids, with larger coverage of the temporal areas. Moreover, the location of the electrodes toward similar positions as those corresponding to the cEEGrid likewise does not produce a significant decrease in decoding accuracy (Figure 3C).

### Effect of the Electrical CI Artifact

Auditory electrophysiological measurements in CI users are corrupted by artifacts introduced by the CI. They can be caused by the radiofrequency transmission between the external part and the implanted part of the CI, or by the electrical stimulation in the cochlea. These artifacts can impact both the scalp and the cEEGrid recordings. A simple physical model based on a melon was used to investigate the effect of the CI artifact on the cEEGrid recordings. Two MED-EL Sonata implants (MED-El, Innsbruck, Austria) were implanted in a melon, simulating in this scenario a head. The headpiece of each CI was placed outside on the melon skin and connected to an OPUS speech processor. In total, two speech processors and two implants were used. The two cEEGrids were placed on each side of the water melon. The melon passed exactly the same procedure as the human subjects. The two audio stories were presented to the speech processors and data were recorded via the cEEGrid. Since melons do not generate any neural activity, any selective attention decoding in the simulation is solely based on the electrical artifact caused by the stimulation of the CI. Since the CI extracts the envelope of the audio signal, it is expected that the selective attention decoding accuracy does not exceed 50%, as both, the attended and the unattended story should result in similar correlation coefficients. Note that the aim of this model is not to model the volume conduction of in a human head, but to demonstrate that selective attention based solely on the CI artifacts is not possible. For a more detailed human model of the volume conduction after CI stimulation refer to Wagner et al. (2018).

**FIGURE 4:**
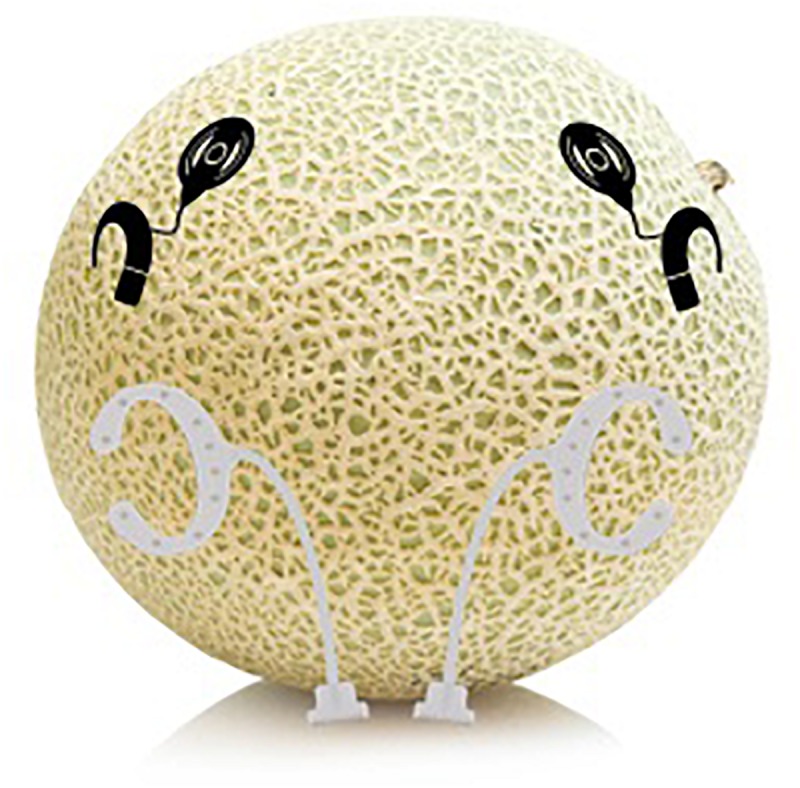
Schematic illustration of the experimental setup to investigate the influence of the cochlear implant (CI) artifact on the decoding accuracy.

**FIGURE 5:**
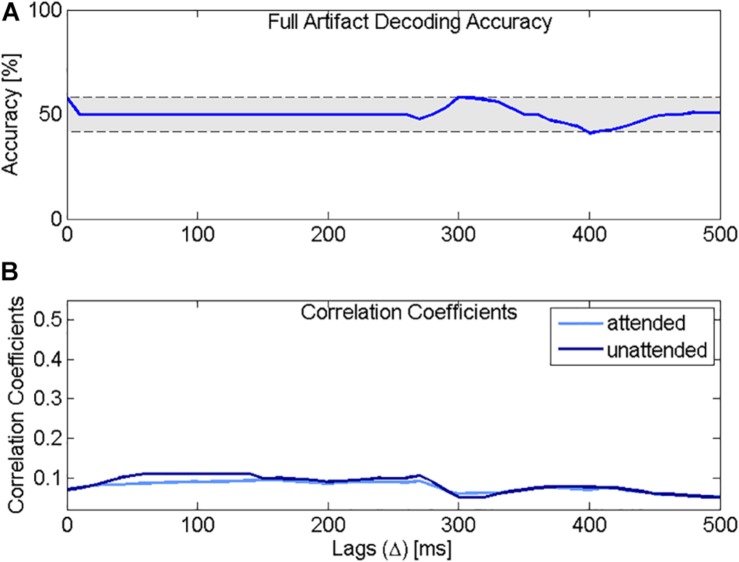
Decoding accuracy and correlation coefficients using the physical model. **(A)** Selective attention accuracy using the attended decoder, trained with a simulated EEG signal containing only artifact. **(B)** Correlation coefficients for the attended speech (light blue) and the unattended speech (dark blue) using the attended decoder trained with simulated EEG data containing only artifact. The correlation coefficients for the attended and the unattended speech sounds overlap and drop rapidly with increasing lag.

Figure 5 presents the decoding accuracy and the correlation coefficients for the attended decoder using the model presented in Figure 4. The results confirm that indeed, if only artifact is present in the EEG recording, the accuracy is below chance level (Figure 5A). Additionally, Figure 5B shows that the correlation coefficients between the reconstructed attended signal and the attended or the unattended speech are very similar.

### Selective Attention Decoding Accuracy Using Individual Regularization Parameters λ

**FIGURE 6:**
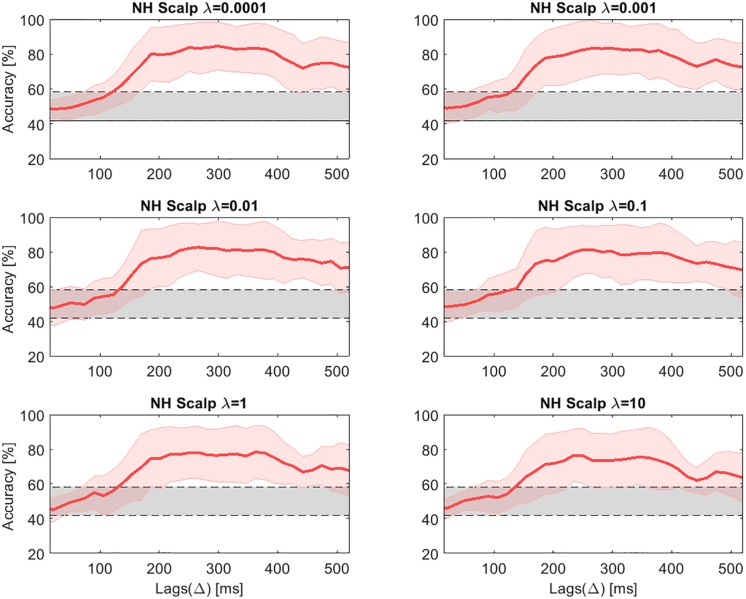
Decoding accuracies in NH listeners for the high-density scalp EEG data across lags for different values of the regularization parameter **λ**. The gray shaded areas show the chance level.

**FIGURE 7:**
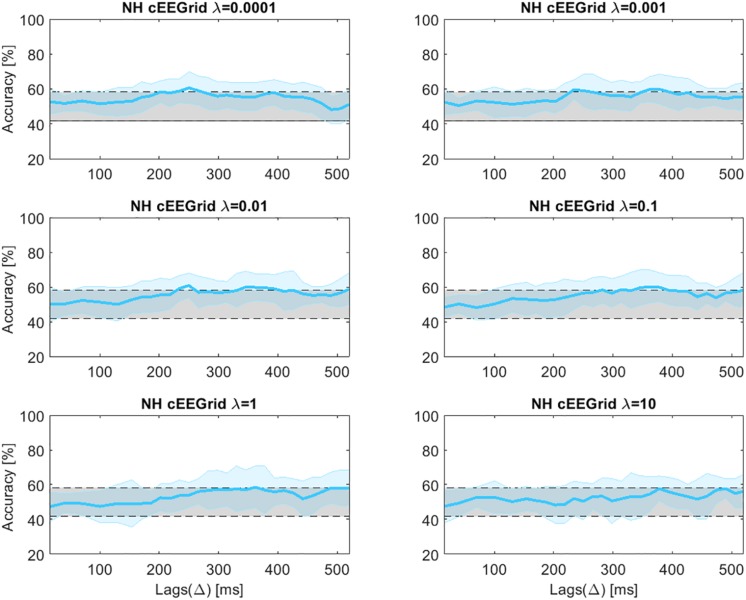
Decoding accuracies in NH listeners for cEEGrid data across lags for different values of the regularization parameter λ. The gray shaded areas show the chance level.

One factor that might have a large impact on decoding selective attention is the regularization parameter λ. For this reason, a parametric analysis was conducted, evaluating the influence of λ on the decoding accuracy. Figures 6, 7 present the decoding accuracies in NH listeners for scalp and cEEGrid data, respectively, using different values of λ (0.0001, 0.001, 0.01, 0.1, 1, and 10). The chance level was estimated as 0.95 of the confidence interval of a binomial distribution and resulted in a range between 41.8 and 58.2% (Combrisson and Jerbi, 2015). For scalp EEG data, the highest accuracy in NH listeners was reached with λ equal to 0.001. The same value (λ = 0.001) obtained the best results for the cEEGrid data in NH listeners. As already pointed out, it may be reasonable to use individual λ values for each CI user, as each subject has received different input from the CIs, depending on the sound coding strategy and the stimulation levels, as well as the specific artifacts caused by the different radiofrequency (RF) transmission systems. Table 3 presents the λ that resulted in the highest accuracy for each CI user. However, unless otherwise specified, the data from the CI group was processed using the same λ value, which coincided with the one found for the NH group.

**TABLE 3:** Individual best λ value for CI users.

**#**	**CI1**	**CI2**	**CI3**	**CI4**	**CI5**	**CI6**	**CI7**	**CI8**	**CI9**	**CI10**
λ Scalp	1	1	0.0001	0.001	1	0.0001	0.001	0.001	0.01	0.001
λ cEEGrid	0.0001	0.01	0.0001	0.01	0.0001	0.01	0.0001	0.1	0.001	0.01

### Correlation Coefficients for Scalp and cEEGrid Data

**FIGURE 8:**
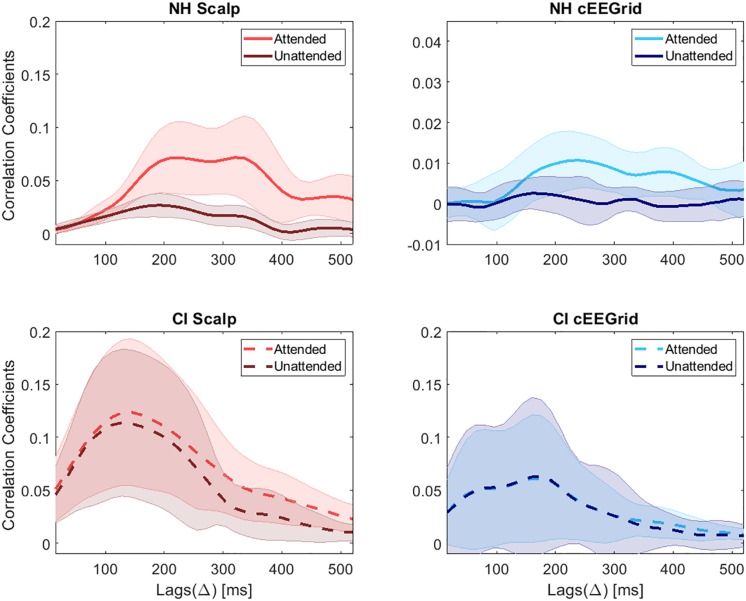
Correlation coefficients for NH listeners and CI users. In each subplot lighter colors refer to the correlation coefficients for the attended speech and darker colors refer to the correlation coefficients for the unattended speech. The thick lines represent the mean values and the shaded areas the standard deviation across subjects.

An attention related difference in the correlation coefficients for the attended and the unattended speech envelope is a precondition for the identification of the attended speaker. Figure 8 presents the correlation coefficients between the original attended and the reconstructed speech, as well as the correlation coefficients between the original unattended and the reconstructed speech, using the attended decoder. The results are presented for each condition (scalp EEG, cEEGrid) and group (NH, CI). The results correspond with averaged correlations across subjects for each lag (Δ). In NH listeners, highest correlation coefficients for the scalp EEG data were obtained in the lag interval of 180–350 ms. These results are in agreement with previous results obtained in the same laboratory (Nogueira et al., 2019). For the cEEGrid data, the interval with highest correlation coefficients in NH listeners was 200–400 ms. The course of correlation coefficients over lags is similar for scalp EEG and cEEGrid data in NH listeners. However, compared to the results of the scalp EEG data, the difference between the attended and the unattended correlation coefficients of the cEEGrid data decreased. For CI users the highest correlation coefficients for the scalp EEG data are observed for lags of 80–250 ms. The largest difference between the attended and the unattended coefficients, however, is observed for lags between 300 and 400 ms, which is delayed with respect to the NH listeners. For the cEEGrid data in CI users, the highest correlation coefficients are observable at even earlier lags from 0 to 250 ms. Moreover, the difference between coefficients for the attended and the unattended speech decreased significantly, with both courses becoming largely overlapping.

In general, correlation coefficients are much larger in CI listeners for both, scalp EEG and cEEGrid data. This difference might be explained by the huge artifact introduced by the CI, which is related to the envelope of the speech. However, with increasing lags, at around 200 ms, the correlation coefficient between the reconstructed attended signal and the original attended speech becomes larger than the correlation coefficient with the unattended sound, at least for scalp EEG data. This, in turn, would enable the decoding of selective attention in CI users at later lags, where the artifact has no strong influence. It seems like the CI artifact correlates only with the speech at short lags. These results agree with the results of our previous study (Nogueira et al., 2019).

It is worth noting that the morphology of the correlation coefficients across lags, using scalp or cEEGrid data was pretty similar. However, the difference between the attended and the unattended correlation coefficients was smaller using the cEEGrid resulting in lower decoding accuracies.

### Overall Decoding Accuracies

**FIGURE 9:**
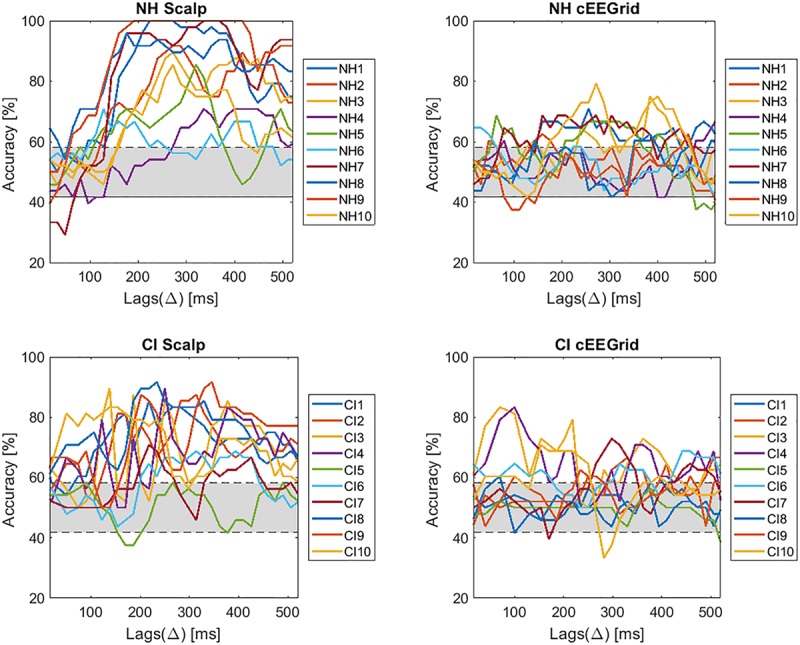
Selective attention accuracies for each subject across lags.

Figure 9 presents the individual single-subject decoding accuracies across lags for NH listeners (top panel) and CI users (bottom panel) for scalp EEG (left panel) and cEEGrid data (right panel).

The individual results demonstrate a large inter-subject variability. All subjects of the NH group obtain accuracies above chance level with the scalp EEG data. However, for the cEEGrid data only 5 out of 10 obtained results significant above chance level. In the group of CI users 9 out of 10 patients show decoding accuracies above chance level when using the scalp EEG data. Performance, accordingly, is almost the same as in NH listeners, which proves that it is possible to decode selective attention in CI users. When using the cEEGrid the performance further dropped. Only 5 out of 10 CI users obtained decoding accuracies above chance level, the same as for NH cEEGrid, but with a lower mean accuracy.

**FIGURE 10:**
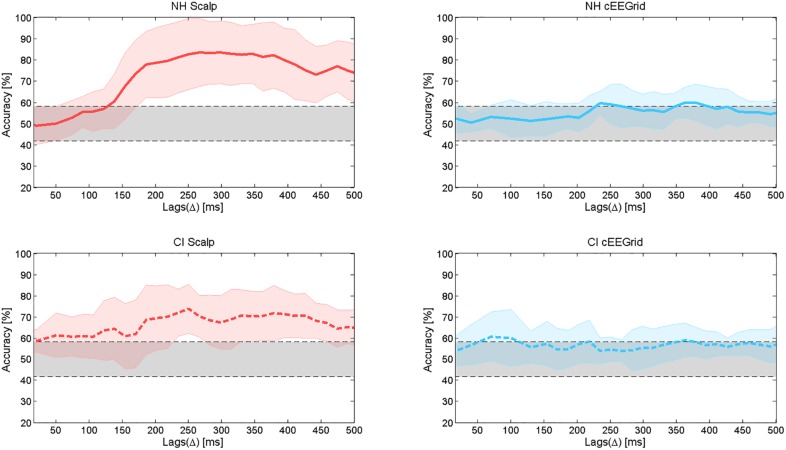
Average decoding accuracies and standard deviation of the mean. The upper panel shows the average decoding accuracies in the group of NH listeners, for both the scalp EEG (left) and the cEEGrid (right) data. The lower panel shows the average decoding accuracies in the group of CI users, for both the scalp EEG (left) and the cEEGrid (right) data.

Average decoding accuracies across NH subjects and across CI users are shown in Figure 10. The accuracy values were obtained using the attended decoder. Chance level (29.2–58.2%) was determined using a binomial test at 5% significance level.

In Figure 8, it can be observed that the correlation coefficient across lags shows two peaks for the scalp EEG data in the group of NH listeners. For this reason, the accuracy values presented in Figure 10 (top left) also show two peaks, one at around 200–260 ms with a maximum value of 82.7% and a second one at around 340–380 ms with a maximum value of 81.04%. For the NH cEEGrid data, these two peaks can also be observed at similar lags but with lower values, that is, 59.58 and 59.79% for the first and second peak, respectively. For the scalp EEG data in the group of CI listeners, the same two peaks can be observed at similar lags with accuracies of 73.8 and 71.67%, respectively. For the CI cEEGrid data, however, the difference between the attended and the unattended correlation coefficient is only visible beyond 350 ms (Figure 8 bottom right). This causes the accuracy to reach the significance level only at around 350 ms (Figure 10 bottom right). Note that a peak in accuracy for the cEEGrid data of CI users can also be observed at 100 ms, however, this peak may be explained by random fluctuations as the attended and the unattended correlation coefficients at these short lags are low and very similar (Figure 8 bottom right).

### Optimized Overall Performance

**FIGURE 11:**
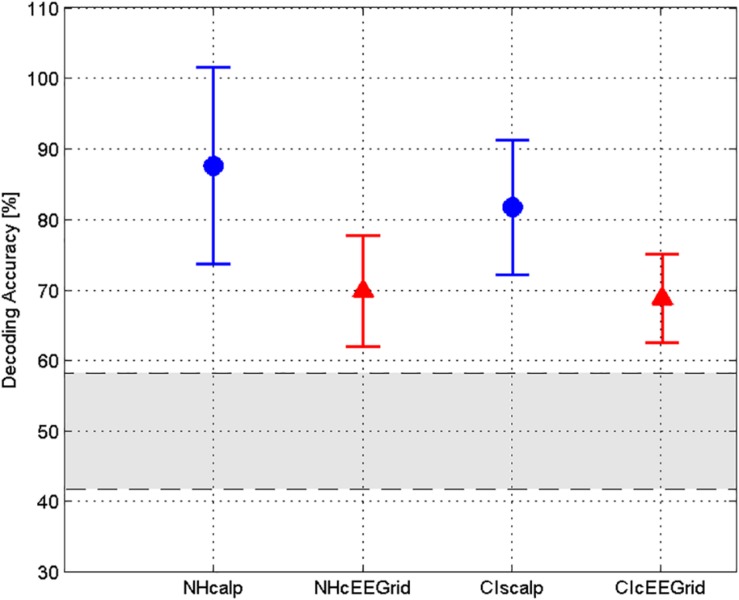
Individually optimized decoding accuracies. Average decoding accuracies for NH listeners and CI users for both, scalp EEG and cEEGrid data. Note, for the CI users the accuracies are obtained using the individual optimal lag and regularization parameter**λ**. The optimal **λ** for each CI subject can be found in Table 3. The gray area indicates the chance level.

Given that each CI user likely obtains quite different CI excitation patterns that, among other factors, depend on the CI manufacturer, the radio frequency link, the sound coding strategy and the fitting parameters, it seems plausible to optimize the decoder on an individual level. Figure 11 presents the decoding accuracies for NH listeners and CI users for both, scalp EEG and cEEGrid data, using for each single CI user the individual optimal lag and regularization parameter λ. Individualizing the regularization parameter and the lag at which the decoding accuracy is estimated results in an increase of the decoding accuracy for CI users using the cEEGrid. Note that the same value of the regularization parameter λ = 0.001 was used for all NH listeners.

The results show that the cEEGrid causes a drop in accuracy with respect to the scalp electrodes and that selective attention decoding is more challenging in CI users than in NH listeners.

## Discussion

This study confirmed previous results that it is possible to decode selective attention in NH listeners using a high-density EEG (Mirkovic et al., 2015; Nogueira et al., 2019), as well as a mobile EEG system with only 18 electrodes (Mirkovic et al., 2016). There has been another study by Fiedler et al. (2017), showing that even only two electrodes – a single in - ear - EEG electrode and an adjacent scalp EEG electrode – are sufficient to identify the attended sound source. Moreover, the present work showed that selective attention can also be decoded with both systems in some CI users, even if the CI introduces large electrical artifacts. However, the inter-subject variability is very high, with not all CI users reaching decoding accuracies above chance level. Generally, the decoding accuracy dropped when using the cEEGrid compared to the high-density scalp EEG data. Nevertheless, it was still just above chance level in both groups.

Our results showed that selective attention in NH listeners using a high-density EEG system can be decoded with a maximum accuracy of 83.3%. These results are consistent with previous studies that reported accuracies of 82–89% based on 30 trials (60 s each) of EEG data recorded from a high number of electrode positions (e.g., O’Sullivan et al., 2015). The results of the present study further confirmed previous findings (Mirkovic et al., 2016), that reducing the amount of scalp electrodes did not have a very strong influence on performance, at least if the number of electrodes is larger than 14. The decoding accuracy using 19 central electrodes around Cz reached a maximum of 80%, while the accuracy using 14 electrodes in temporal regions close to the ears reached 83%. These results are in agreement with previous findings (Mirkovic et al., 2016) and suggest that it is possible to decode selective attention with around-the-ear electrodes such as with the cEEGrid. It should be noted, however, that the channels we have chosen for the comparative analysis with the cEEGrid spatially reach a wider area than the cEEGrid arrays, potentially covering the auditory cortex and thus capturing the dipole resulting from auditory activity to a greater extent. This might be the reason why in the present study, the difference between the simulated around-the-ear decoding accuracy and the cEEGrid decoding accuracy was bigger than 20% in NH listeners. The cEEGrid decoding accuracy is slightly below the maximum decoding accuracy (69.3%) found by Mirkovic et al. (2016).

The present work was based on a previous study, using a highly similar paradigm and decoder to assess and compare the decoding of selective attention with the cEEGrid system and a high-density EEG system in NH listeners (Mirkovic et al., 2016). In the study by Mirkovic et al. (2016) the stories were always presented dichotically but on the same side. In contrast, the current study switched the side of attention from trial to trial. In the study by Mirkovic et al. (2016), the maximal accuracies were 84.78% for the high-density EEG system and 69.33% for the cEEGrid. The accuracy reached with the high-density EEG electrodes was similar to the accuracy obtained in the present study in NH listeners. However, the accuracy obtained with the cEEGrid was around 6% higher than in the current study. Only two of their 18 NH participants did not reach accuracies above chance level, whereas in the current study only 5 out of 10 NH subjects reached accuracies above chance level with the cEEGrid. Another difference between both studies is the age of the participants, 24.8 years vs. 42.8 years.

The large difference between the cEEGrid performance and the scalp EEG performance using a decreased number of electrodes can be explained by the position of the reference electrode. The cEEGrid uses a local reference that is the reference, the ground and the recording electrodes are all allocated around-the-ear, which gives small signal amplitudes. For the scalp EEG using a decreased number of electrodes around-the-ear, however, the nose tip is used as reference, which is located far away from the recording electrodes and therefore gives higher amplitudes. Apparently this amplitude/signal to noise ratio is crucial for good decoding (Bleichner and Debener, 2017).

Somers et al. (2019) showed that it is possible to perform neural tracking of the speech envelope in CI users if care is taken about the artifacts introduced by the CI. The study applied an approach that periodically interrupted the electrical stimulation of the CI. In these gaps, the EEG signal was recorded without any artifact. This work was extended by Nogueira et al. (2019), who showed that the decoding using high-density scalp EEG is as well possible without stimulation gaps, in the presence of a continuous electrical artifact. We obtained an average decoding accuracy across subjects of 70.5% (Nogueira et al., 2019). In the present study we obtained a similar decoding accuracy of 73.8%. Analyzing the data on an individual level shows that only one subject out of 10 obtained a decoding accuracy below chance level when using the high-density EEG system. Using the cEEGrid the performance, however, dropped and reached an average decoding accuracy of 59.79%, which is just above chance level. On an individual level, only five subjects achieved decoding accuracies above chance level.

The decoding accuracy was analyzed across lags to better characterize the physical delay between the presented sounds and the recorded neural responses. Previous studies on cortical responses to attended continuous speech in NH listeners have reported peak latencies or lags of the highest decoding accuracy ranging from short delays (100 to 150 ms; Ding and Simon, 2012; Koskinen and Seppä, 2014) to longer delays (150–300 ms; e.g., Aiken and Picton, 2008; O’Sullivan et al., 2015; Mirkovic et al., 2016). NH listeners (scalp and cEEGrid) in the present study showed the maximum decoding accuracy at lags of around 260 ms, which is consistent with these previous findings. The data, both, for scalp EEG and cEEGrid, however, actually showed two peaks in accuracy, the first one at 200–260 ms and the second one at 340–380 ms. For CI users only the high-density scalp EEG, but not the cEEGrid data showed two peaks in decoding accuracy occurring at similar lags as in normal hearing listeners. For the cEEGrid data in CI users, the accuracy remained below chance level for most of the lags. At 350 ms (corresponding with the second peak in the other datasets), however, the accuracy just reached significance. Another peak in accuracy at around 100 ms was observed, but correlation coefficients at this lag were very low and very similar to each other, and for this reason this early peak is attributed to random fluctuations.

The methodology used in the present study uses backward mapping of neural responses to reconstruct the speech envelope. This mapping approach derives a reverse stimulus-response mapping by exploiting all of the available neural data in a multivariate context (Haufe et al., 2014; Crosse et al., 2016). In contrast to the forward mapping approach, with the backward model the decoder channel weights are not readily interpretable in a neurophysiological sense, their weighting reflects the channels that contribute most toward reconstructing the stimulus signal (Haufe et al., 2014). Previous studies have shown that using all EEG electrodes to reconstruct the speech envelope results in the highest decoding accuracy, however the number of EEG electrodes can be reduced to 25 without decreasing performance (e.g., Mirkovic et al., 2016). In Nogueira et al. (2019), we showed the power spectral density of each EEG electrode across frequency and location. In CIs, the highest power spectral density was observed at locations close to the CI electrodes resulting in higher values for the decoder weights corresponding to these locations.

One possibility to improve the accuracy in CI users would be to use artifact rejection methods such as independent component analysis relying on covariance of multiple EEG channels (Makeig et al., 2004). Moreover, new artifact rejection algorithms, especially algorithms designed to remove the artifact of the CI, combined with sound coding strategies that reduce the CI artifact (Somers et al., 2019) may optimize selective attention decoding accuracy. Another possibility to improve the decoding accuracy would be based on novel decoding algorithms. Most auditory attention detection algorithms use a linear EEG decoder to reconstruct the attended stimulus envelope. Classifying attention within a short time interval remains the main challenge. However, since the human auditory system is inherently non-linear (Faure and Korn, 2001), non-linear models could be beneficial. Recent developments have shown that convolutional neural networks (CNN) can be used to solve this problem (Deckers et al., 2018; Taillez et al., 2018).

The application of high-density EEG systems in the clinical environment is highly limited, as preparation time is fairly long. Moreover, the use of high-density EEG-systems for daily life applications is unrealistic. Here, miniaturized EEG systems, located around-the-ear or even within-the-ear, would constitute a clear advantage. In parallel, it is necessary to develop optimized paradigms to objectively quantify speech performance with hearing devices to be able to automatically fit and adapt the respective signal processors. In this context, it would be interesting to investigate whether the accuracy in decoding selective attention or the correlation coefficient between the reconstructed and the attended speech indeed correlate with the subject’s speech performance in larger cohorts.

The implementation of unobtrusive recording functionality seems possible by adding EEG recording functionality into a future CI, by attaching a few electrodes in the outer ear canal (Bleichner et al., 2015; Mikkelsen et al., 2015) or by placing electrodes around the ear (Debener et al., 2015; Mirkovic et al., 2016). Since EEG measures voltage, that is, the potential difference between two locations, the exact position of the electrodes, the distance between the electrodes and the orientation of the two electrodes relative to the source signal of interest, influences whether a signal of interest can be captured. Current technology is far from optimal for monitoring signals reflecting selective attention. However, designing a closed-loop CI that integrates physiological signals into this loop seems feasible. It has been demonstrated that by using the extra-cochlear electrode in a CI as a recording electrode it is possible to record longer latency neural responses and that the timings of these responses are in general agreement with auditory brainstem and auditory cortex responses recorded with scalp electrodes (McLaughlin et al., 2012). In the near future, it therefore might be possible to decode selective attention from CI electrodes without the need of additional external electrodes.

In general, a classification accuracy of around 70% after 1 min of training might not yet comply with the requirements of a CI user to steer sound processing algorithms in a cocktail-party like scenario. Furthermore, the current measurements did not resemble real life situations. The data were recorded in a shielded room with reduced environmental noise. Moreover, subjects were asked to move as less as possible to minimize muscle artifacts. The current study has used a very simple paradigm to simulate the cocktail party effect. The results presented here are therefore not easily applicable to daily life situations and more realistic sound environments (e.g., Das et al., 2016) need to be explored to better understand the selective attention processes in both NH and CI users. Nevertheless, the use of novel algorithms to decode selective attention, EEG artifact rejection methods and the use of implanted electrodes, such as the ones employed in a CI may held potential for a future closed-loop system.

## Conclusion

The current work was able to confirm that it is possible to decode selective attention in NH listeners with high decoding accuracies using a high-density scalp EEG, as well as a small mobile EEG system, in this case consisting of 2 cEEGrid arrays, each containing 10 electrode contacts. However, the decoding accuracy was significantly lower with the cEEGrid, compared to the high-density scalp EEG. Furthermore, the study could show that it is possible to decode selective attention in CI users despite of the introduced electrical artifact. Though, the inter-individual variability in CI users is high and when using the mobile EEG system, the decoding accuracy dropped significantly, being just above chance level on the group level. However, results on the single-subject level showed that in several CI users selective attention could be successfully decoded with cEEGrids. An individual optimization of certain parameters of the algorithm, as the regularization parameter or the best time lag, further improved the decoding accuracy.

## Ethics Statement

Ethics Committee of the Hannover Medical School (Study ID: 3229-2016).

## Author Contributions

WN conceived the study, implemented the algorithm, conducted the data collection and analysis, and wrote the manuscript. HD and IS conducted the data collection and analysis, and contributed to writing of the manuscript. BM contributed to experimental setup, advised on the data analysis, and contributed to writing of the manuscript. AB contributed to writing of the manuscript. MB advised on experimental setup and contributed to writing of the manuscript. SD contributed to writing of the manuscript, and advised on the data analysis and interpretation.

## Conflict of Interest Statement

The authors declare that the research was conducted in the absence of any commercial or financial relationships that could be construed as a potential conflict of interest.

## Footnotes

1 https://github.com/sccn/labstreaminglayer
